# From early defence priming to lasting memory: developmental and seasonal dynamics in trees

**DOI:** 10.1042/EBC20250063

**Published:** 2026-07-30

**Authors:** Melissa Mageroy, Estrella Luna, Adriana Puentes

**Affiliations:** 1 Norwegian Institute of Bioeconomy Research, Division of Plant health and biotechnology, Ås 1432, Norway; 2 Birmingham Institute of Forest Research, School of Biosciences, University of Birmingham, Birmingham B15 2TT, U.K.; 3 Department of Ecology, Swedish University of Agricultural Sciences (SLU), Box 7044, Uppsala, SE-75007, Sweden

**Keywords:** Defense priming, Long-lived trees, Ontogeny, Stress memory

## Abstract

Defence priming enhances plant responsiveness to future stress following prior exposure, and has been extensively characterised in annual model species as a reversible state associated with faster or stronger defence activation. However, studies in long-lived plants indicate that priming responses can be elicitor-specific, developmentally variable and closely linked to environmental history. In trees, priming is often strongest in early life stages, while in mature individuals defence responses are more frequently associated with direct activation and stabilised regulatory states. Evidence across systems shows that priming can operate over multiple timescales, from short-term reversible responses to longer-lasting effects that influence response thresholds and activation kinetics. Epigenetic mechanisms, including DNA methylation and chromatin modification, contribute to these processes and may persist across cell divisions or developmental transitions. Together, these findings highlight the importance of developmental stage and temporal context in shaping plant defence responses.

## Introduction: priming revisited

Defence priming is increasingly recognised as an important mechanism through which plants adjust their responsiveness to environmental stress [1–4]. Rather than constitutively activating costly defence responses, plants can enter a primed state following prior exposure to biotic or abiotic cues, allowing them to respond more rapidly or more strongly when stress recurs. Much of our current understanding of defence priming comes from work in annual model plants and crop systems [2,3]. In these species, priming is often conceptualised as a preparatory physiological state that enhances inducible immunity without constitutive defence activation [1]. Mechanistically, this enhanced responsiveness has been linked to a variety of regulatory processes, including transcriptional reprogramming, chromatin remodelling, metabolic changes and signalling network reconfiguration [5–8]. Priming in annual plants is frequently interpreted through well-characterised defence signalling pathways, including salicylic acid (SA)- and jasmonic acid (JA)-mediated responses, and is often treated as a paradigm of plant acquired resistance [9]. This capacity to modulate future responses based on past experience represents a form of immunological memory and contributes to the remarkable plasticity that enables plants to cope with fluctuating environments [2].

However, emerging evidence from long-lived plants suggests that this model may not fully capture the diversity of priming regulation across plant life histories. Trees encounter repeated cycles of biotic and abiotic stress over decades or centuries. Under this pressure, immune regulation must integrate past environmental experience with future preparedness over far longer timescales than those typically studied in annual plants [10]. Increasingly, studies in woody species indicate that defence regulation can differ from patterns described in herbaceous models [7,11,12]. For example, recent work in oak seedlings (*Quercus robur*) shows that priming can be elicitor-specific, with chemically distinct stimuli triggering distinct defence responses and regulatory signatures that involve different combinations of transcriptional activation, structural defence deployment and signalling pathway engagement [13]. Additionally, studies including Norway spruce (*Picea abies*) indicate regulatory machinery, signalling cross-talk and interpretation of regulatory markers may differ in these long-lived, evolutionarily distinct trees [14]. In these systems, immune responses appear embedded within regulatory networks shaped by long life spans, repeated stress exposure, and complex developmental trajectories. Such conditions may favour immune strategies that integrate environmental history over extended periods, resulting in defence responses that differ in timing, flexibility, and regulatory control from those typically described in short-lived herbaceous species [10].

In the present review, we explore how defence priming and stress memory are expressed across developmental stages in long-lived trees, from embryogenesis and seedlings to mature individuals. We focus on how the timing of stress exposure within the life cycle and across seasons influences the establishment, maintenance, and resetting of primed states, with particular emphasis on epigenetic regulation and dormancy-related reprogramming. Within this framework, we treat short-term seasonal priming and longer-lasting stress memory as a temporal continuum and propose that ontogeny functions as a regulatory filter that governs which priming effects persist, are reinforced, or are erased across development.

## Epigenetic foundations of priming in long-lived plants

**Figure 1:**
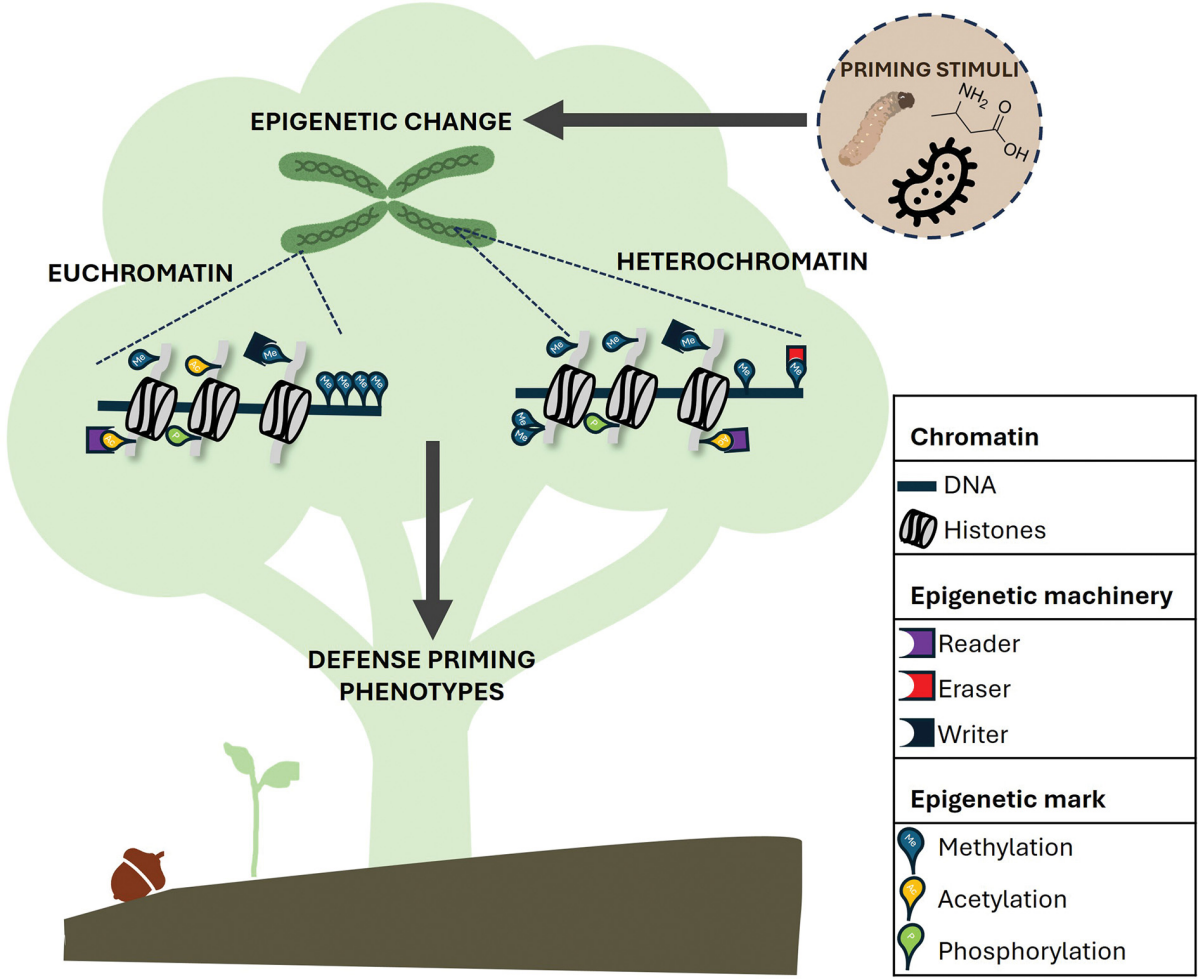
Epigenetic regulation of stress memory and defence priming in trees Chromatin-based stress memory in trees. Biotic and abiotic stimuli in the environment trigger changes in the chromatin state at specific genomic regions, including defence-related genes. These changes occur through modifications of epigenetic marks on DNA and histones, carried out by epigenetic machinery such as writers, readers, and erasers. Altered chromatin states lead to changes in gene expression. Chromatin that is open and accessible for transcription is called euchromatin, whereas compact and transcriptionally inactive regions are termed heterochromatin. The resulting shifts in gene accessibility contribute to defence-priming phenotypes. The formation and persistence of stress memories can vary across different developmental stages of a tree.

Increasing evidence indicates that both priming and stress memory involve epigenetic regulation, where previous stress experience alters gene activity without changing the underlying DNA sequence. Chromatin-based mechanisms can maintain stress-responsive genes in a more permissive transcriptional state, allowing faster or stronger activation during subsequent challenges (Figure 1). Several epigenetic regulatory layers contribute to this process, including DNA methylation, histone modifications, and small non-coding RNAs, which together shape chromatin accessibility and transcriptional potential [10] (Figure 1). In plants, DNA methylation occurs in CG, CHG, and CHH sequence contexts, where the letters indicate the identity of nucleotides surrounding the methylated cytosine (C). In CG contexts, a cytosine is followed by a guanine; in CHG and CHH contexts, H represents any nucleotide except guanine (adenine, thymine, or cytosine), meaning that CHG sites have a symmetric arrangement (C followed by a non-guanine base and then a guanine), whereas CHH sites are asymmetric. These different contexts are maintained and regulated by distinct enzymatic pathways and can differ in their stability and responsiveness to environmental cues [15]. Changes in methylation across these contexts are often associated with altered chromatin compaction and gene expression. Moreover, histone tail modifications such as acetylation and methylation provide an additional dynamic layer of transcriptional control by altering chromatin structure and regulating the accessibility of DNA to the transcriptional machinery [16,17] (Figure 1). For example, histone acetylation is generally associated with a more open chromatin configuration and active gene expression, whereas specific histone methylation marks can either activate or repress transcription depending on their position and context [16]. These modifications are reversible and can be rapidly established or removed in response to environmental cues, making them particularly well suited to support priming-related changes in gene responsiveness. Importantly, some epigenetic marks can persist through mitotic cell divisions, allowing priming effects to last weeks to months, and in certain cases may be transmitted through meiosis, contributing to intergenerational or transgenerational stress memory [10,18] (Figure 1).

This epigenetic dimension is particularly relevant in long-lived plants. Trees experience decades of environmental variability and repeated cycles of biotic and abiotic stress, requiring regulatory systems capable of integrating environmental history across extended lifespans while maintaining developmental coordination [14,19,20]. Seasonal fluctuations play a central role in structuring these responses, synchronising growth, dormancy, and stress physiology, and increasingly appear to be underpinned by epigenetic regulation [21]. Recent work in mature oak (*Q. robur*) provides direct evidence that DNA methylation landscapes are dynamically remodelled across seasons, with progressive changes in CHH methylation from spring to summer and autumn, indicative of ongoing epigenetic reprogramming within a single growing cycle [22]. These seasonal shifts are associated with differential methylation of promoter regions and transposable elements, as well as genes involved in leaf development and signalling pathways, suggesting that epigenetic regulation is tightly linked to both phenology and environmental responsiveness.

At the same time, comparisons between mature trees and their progeny reveal distinct generational methylation signatures, particularly in CG and CHG contexts within genic regions, pointing to a developmental and heritable dimension of epigenetic regulation [23]. Together, these findings highlight that epigenetic states in trees are not static but are continuously reshaped across both seasonal and developmental timescales. This capacity for dynamic yet structured epigenetic modulation provides a mechanistic basis for adaptive phenotypic plasticity, allowing trees to respond to recurring environmental challenges while progressively stabilising aspects of immune and developmental regulation as ontogeny advances. In this context, epigenetic mechanisms offer a means by which environmental experience can be integrated, filtered, and, in some cases, retained, linking short-term physiological responses with longer-term trajectories of plant performance and resilience [14].

## Ontogeny as a developmental filter for priming

**Figure 2:**
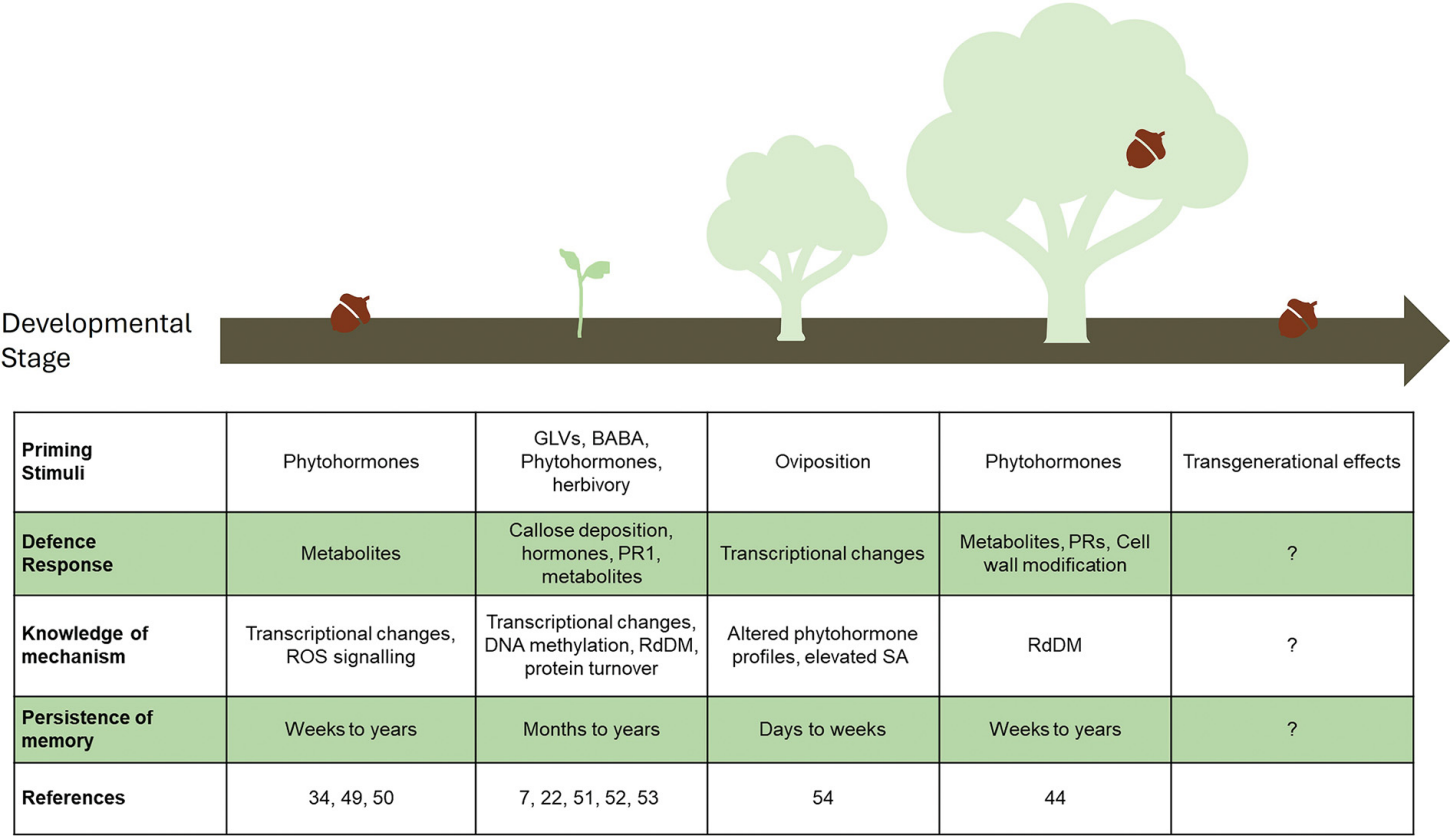
Defence priming across tree development Examples of priming stimuli, defence responses, underlying mechanisms, and memory persistence at each tree developmental stage [49–54]. Early developmental stages (e.g., seedlings and young saplings) are among the most studied because they are easier to handle experimentally and allow shorter study durations. In contrast, very little information is available for later life stages, especially mature trees, and transgenerational effects, due to the long time required for experiments, large plant size, and logistical limitations. As a result, knowledge about priming mechanisms and the duration of induced memory in adult trees is still limited and often speculative. References supporting current knowledge are shown in the table. BABA: β-aminobutyric acid; GLVs: green leaf volatiles; PR: pathogenesis-related protein; RdDM: RNA-directed DNA methylation; ROS: reactive oxygen species.

Priming competence is not fixed but varies across the plant life cycle, indicating that the capacity to establish, maintain, and express primed states is developmentally regulated (Figure 2). In tree species, immune priming is typically most easily detected and experimentally tractable during early life stages, particularly in seedlings, while the persistence of primed states can be strongest at the very earliest stages such as embryogenesis. In mature trees, induced responses are often more transient or less clearly distinguishable from direct defence activation. This suggests that priming is not uniformly expressed across ontogeny, but instead reflects changes in the regulatory architecture of plant immunity over time. Importantly, this developmental shift does not imply a loss of defence capacity. Mature trees retain extensive defence gene repertoires [24] and can mount strong responses to biotic stress. However, their immune behaviour appears increasingly governed by regulatory states shaped by cumulative environmental exposure and developmental history. As a result, the flexibility associated with inducible priming responses may decline with age, while defence regulation becomes progressively stabilised.

Defence priming provides plants with a powerful mechanism to adjust immune behaviour within a lifetime by linking short-term plastic responses to the selective persistence of stress memory. Rather than replacing genetic adaptation, priming enables improved phenotypic matching to recurring environments, allowing plants to optimise defence deployment in response to predictable biotic and abiotic challenges [10]. This capacity is particularly important for long-lived species, which experience repeated stress cycles across seasons and developmental stages and must balance defence with growth over extended timescales.

Evidence from annual plants supports the idea that priming can be established early and persist across substantial portions of the life cycle, for example, following seed treatments that induce long-lasting resistance to later pests and pathogens [2,25,26]. Whether this is similar in trees or not remains unexplored; however, one could hypothesise that in long-lived trees, repeated exposure to environmental variability and recurrent immune activation may progressively reshape defence regulation, shifting it away from highly flexible priming states towards configurations that reflect accumulated stress experience. As a consequence, priming in mature trees may not manifest as a distinct state, instead reflected in altered response thresholds.

### Embryo and early development

Early ontogeny is characterised by high developmental plasticity and extensive regulatory reprogramming, creating a window during which environmental cues can have disproportionate and long-lasting effects on plant phenotype [27]. This aligns with broader concepts of plant environmental memory, where early life experiences can influence later developmental stages if regulatory states persist through ontogenetic transitions. In long-lived species, epigenetic reprogramming during embryogenesis provides a potential mechanism for such effects. For example, temperature conditions during embryo development have been shown to generate persistent ‘epitypes’ associated with long-term changes in gene expression, growth patterns, and phenology [14,28–30]. Similarly, exposure to phytohormones or stress-related cues during somatic embryogenesis can induce lasting changes in defence-related traits. In Norway spruce, somatic embryo-derived plants exposed to defence-inducing conditions show increased resistance to bark-feeding insects [31,32], while in Holm oak (*Quercus ilex*), somatic embryogenesis under stress conditions enhances resistance to the oomycete *Phytophthora cinnamomi* [33]. These findings suggest that early developmental stages may provide a critical window for the establishment of priming-compatible regulatory states, some of which may persist beyond embryogenesis and influence later defence responsiveness. Seed treatment with phytohormones has also been shown to enhance the resistance of Norway spruce (*P. abies*) seedlings to *Botrytis cinerea*, generating a stress memory that may persist for years and may confer broader, multi-stress tolerance [8] (Figure 2).

### Seedlings and juveniles

Seedling stages often represent a peak in priming responsiveness, where inducible defence plasticity is both strong and experimentally tractable [6,26]. In these early stages, plants must rapidly adjust to fluctuating environmental conditions while balancing growth and defence, and priming provides an efficient mechanism to enhance responsiveness without sustained metabolic cost.

In tree seedlings, priming responses are often elicitor-specific and mechanistically diverse, reflecting multiple regulatory pathways that can be engaged depending on the nature of the stimulus (Figure 2). For example, in oak seedlings (*Q. robur*), distinct elicitors can trigger different combinations of defence outputs, including structural responses such as callose deposition or transcriptional activation of defence-associated pathways [13]. Similarly, application of methyl jasmonate in conifer seedlings, including *Pinus* and *Picea* species, induces rapid morphological and chemical changes within a single growing season, enhancing resistance to herbivores and pathogens [34]. Recent work in European ash (*Fraxinus excelsior*) further illustrates both the potential and limitations of priming at this developmental stage. Treatment with the priming agent β-aminobutyric acid (BABA) enhances tolerance to ash dieback caused by *Hymenoscyphus fraxineus*, reducing necrosis development in seedlings several months after inoculation [35]. These responses are accompanied by detectable epigenetic changes, including increased levels of modified cytosine derivatives such as 5-(hydroxymethyl)-2′-deoxycytidine and 5-(hydroxymethyl)-2′-deoxyuridine, suggesting that priming in seedlings can involve measurable alterations in DNA modification states. Correspondingly, in another study, it was found that pre-inoculation of European ash saplings with a low-virulent *H*. *fraxineus* strain primed defences and reduced necrosis expansion by 53% upon subsequent challenge with a highly virulent strain [36].

Together, these findings support the idea that priming in early developmental stages can couple enhanced stress tolerance with epigenetic reconfiguration. Although priming responses at the seedling stage can incur short-term growth costs, these are often transient. In several *Picea* and *Pinus* species, for instance, methyl jasmonate treatment may reduce height growth in the short term, but these effects typically diminish over subsequent years [37]. This suggests that seedlings operate within a regulatory regime that favours responsiveness and flexibility, allowing priming to be expressed strongly when environmental conditions demand it, even if such responses are not always maintained over longer timescales. Notably, despite the conceptual importance of this developmental window, there is a striking lack of studies explicitly addressing priming responses in juvenile trees beyond the seedling stage [34] (Figure 2). This gap limits our ability to understand how priming is maintained, modified, or lost during early ontogenetic transitions and represents a critical area for future research.

### Mature plants

As trees mature, defence strategies are not necessarily constrained by resource limitation in the same way as in early life stages. Seedlings, operating under restricted resource availability, must tightly balance growth and defence, often favouring highly regulated and efficient inducible responses [38]. For example, ontogenetic studies in *Eucalyptus froggattii* demonstrate directionally opposite trajectories for different defense classes. Inexpensive phenolic defenses are maximally deployed in seedlings but decline in mature trees, whereas costly terpenoid defenses requiring specialised secretory structures increase progressively with tree age [39]. Mature trees can, under favourable conditions, allocate substantial resources to defence in a given year, provided long-term survival is maintained [40]. This greater buffering capacity may allow for stronger or more sustained defence activation but may not necessarily translate into increased priming responsiveness.

Differences across ontogeny may also reflect shifts in how defence responses are regulated rather than simple changes in resource availability. Early developmental stages, including seeds and seedlings, are characterised by highly dynamic regulatory processes, in which multiple physiological outputs are coordinated through hormone signalling networks and their interactions [41–43]. This includes extensive cross-talk between pathways such as SA and JA, which are known to shape defence responses in a context-dependent manner. Given the prominence of these signalling dynamics during early development, it is plausible that priming in seedlings relies more strongly on hormone-mediated regulation and cross-talk. Whereas in later life stages, defence responses may be less dependent on transient signalling configurations and more influenced by developmental state and prior exposure.

Empirical evidence from conifer systems illustrates that, in both seedlings and mature trees, elicitors such as methyl jasmonate can induce a range of defence responses, including direct activation, prolonged up-regulation of defence pathways, and priming-like effects. In Norway spruce (*P. abies*), methyl jasmonate treatment enhances resistance to bark beetle attack, including *Ips typographus*, through increased production of defensive compounds and structural barriers [44]. However, distinguishing between priming and direct defence activation remains challenging, as responses can vary with elicitor concentration and tissue properties such as bark thickness, which may influence penetration and responsiveness. This highlights a broader limitation in interpreting priming in mature trees, where induced responses often blur the distinction between activation and sensitisation. Similarly, studies on resistance to the pine weevil (*Hylobius abietis*) in *Pinus* species demonstrate that defence phenotypes in trees reflect a combination of constitutive and inducible traits shaped over time, including chemical and physical defence components [45]. These findings suggest that, in mature trees, defence responses are embedded within a broader physiological and developmental context, where prior exposure, growth patterns, and tissue differentiation influence how defence is deployed.

Long-term experimental and meta-analytical studies further show that repeated application of elicitors such as methyl jasmonate can enhance resistance across multiple conifer species, albeit often with associated trade-offs in growth and resource allocation [34]. Rather than indicating the establishment of a stable primed state, these results suggest that defence enhancement in mature trees may rely on repeated or sustained stimulation, raising questions about the persistence of priming across time and developmental transitions. Across ontogeny, therefore, the key shift may not be from priming to its absence, but from highly flexible, signalling-driven responses towards configurations that are increasingly shaped by developmental state, tissue structure, and accumulated environmental exposure. Evidence from transitions between juvenile and more mature growth phases, including shifts in growth patterns and growth cessation dynamics, further supports the idea that developmental stage influences how environmental signals are integrated and expressed [46]. Within this context, priming in mature trees may be less evident as a discrete state and instead reflected in changes in response thresholds, activation kinetics, or sensitivity to subsequent stimuli. These responses do not necessarily arise from stable, long-term memory in the strict sense but from the interaction between current physiological state, developmental context, and prior exposure history.

Taken together, these observations reinforce the view that priming is a developmentally modulated process. Early life stages favour highly plastic, priming-compatible configurations that enable rapid adjustment to fluctuating environments, whereas later stages reflect a more integrated form of responsiveness shaped by growth, structure, and environmental history. Ontogeny therefore plays a central role in determining how priming is expressed, maintained, or lost, highlighting the need to interpret priming within the broader context of plant development rather than as a uniform physiological phenomenon (Figure 2). Notably, we found that comparable studies of priming in mature non-conifer trees are mostly absent, which was also highlighted in earlier reviews [47,48] and warrants further investigation.

## Seasonal priming as a reversible layer of immune plasticity

Seasonal priming in trees represents a short-term, reversible adjustment of physiological and epigenetic states that align closely with recurring environmental cues. This responsiveness is tightly linked to the dynamics of the dormancy cycle, where changes in photoperiod and temperature regulate the induction, maintenance, and release of endodormancy, particularly in seedlings and juvenile trees that display heightened seasonal sensitivity [55]. Within this framework, seasonal priming can be viewed as part of a broader regulatory system that synchronises growth, stress responsiveness, and developmental transitions across the annual cycle.

Conceptually, such reversible priming aligns with the framework of meristem quiescence, in which dormancy acts as a seasonal checkpoint that allows trees to reset developmental and physiological states in response to shifting and often unpredictable environmental conditions [56]. This annual resetting is underpinned by dynamic epigenetic regulation. In angiosperms, genome-wide analyses reveal seasonal fluctuations in DNA methylation, including changes in CHH methylation associated with bud burst and leaf phenology that are re-established each winter [22]. Similarly, in *Populus*, transitions into and out of dormancy involve large-scale and reversible changes in chromatin accessibility and histone modifications such as H3K27me3, reinforcing the idea that dormancy represents a central, annually reconfigured regulatory hub [57].

In conifers, seasonal responsiveness is further modulated by longer-term epigenetic influences established during early development. Climatic conditions experienced during embryogenesis can imprint persistent DNA methylation patterns that influence traits such as bud burst timing, frost tolerance, and cold-responsive gene expression later in life [14]. The interaction between these longer-term epigenetic imprints and annually resetting seasonal signals enables trees to integrate past and present environmental information, generating the phenotypic plasticity required to cope with interannual variability in climate, disease pressure, and abiotic stress.

Despite this capacity for dynamic adjustment, seasonal priming appears inherently temporally constrained. Primed states associated with seasonal cues are typically reversible and reset during dormancy or major developmental transitions [58]. Evidence from priming studies supports this limitation: for example, treatments with priming agents such as BABA can enhance stress tolerance over short timescales, but the conditions required to establish longer-lasting or developmentally stable effects remain unclear [13,35]. This suggests that, while priming can effectively enhance responsiveness within a given season, its persistence across developmental transitions is not guaranteed.

Taken together, these observations support the view that seasonal priming represents a labile layer of immune regulation that operates within defined temporal boundaries. It enables trees to fine-tune defence responses to predictable environmental cycles but is typically reset before it can transition into longer-term memory. Within the broader temporal framework proposed in the present review, seasonal priming therefore occupies the short-term end of the continuum, providing flexible within-season optimisation, while only a subset of regulatory states may be retained and stabilised as longer-lasting stress memory across developmental time.

## Implications and open questions

A central challenge emerging from current research is to understand when and how priming transitions from a transient, reversible state to a more developmentally stabilised form of stress memory. Although epigenetic mechanisms are frequently invoked as carriers of such memory, distinguishing causal regulatory features from correlated molecular signatures remains a major limitation. This raises fundamental questions about which regulatory layers truly encode past environmental experience and under what conditions these states are maintained, modified, or reset.

Increasingly, it is becoming clear that priming cannot be understood independently of developmental context. Ontogeny shapes not only the capacity to establish priming but also its persistence across time. Early-life stages, characterised by high plasticity and extensive regulatory reprogramming, may provide critical windows during which environmental cues have disproportionate and long-lasting effects. However, whether priming events occurring at these stages are more likely to be retained, reinforced, or erased during subsequent developmental transitions remains unresolved.

Similarly, the predictability and recurrence of stress are likely to play a key role in determining whether priming is maintained or dissipates. In environments where stress is frequent or cyclical, selective pressures may favour the retention or reinforcement of primed states. In contrast, under more stochastic conditions, reversible and transient responses may be more advantageous, reducing the risk of maladaptive defence deployment. Understanding how plants balance these trade-offs is essential for interpreting when priming contributes to longer-term memory versus short-term optimisation.

Addressing these questions will require a shift in experimental design. Rather than treating developmental stage, seasonal context, and prior exposure as sources of variability, they must be explicitly incorporated as central variables. This includes tracking defence phenotypes, molecular states, and regulatory configurations across life stages, seasons, and environmental transitions. Such approaches will be necessary to disentangle the temporal dynamics of priming, identify the conditions under which memory is stabilised, and ultimately understand how plants integrate environmental experience across their lifetimes.

## Summary

- Defence priming in trees forms a temporal continuum, from short-term seasonal priming to longer-lasting stress memory.
- Priming capacity is shaped by ontogeny, being highly flexible in early stages and more stabilised and threshold-based in mature trees.
- Epigenetic mechanisms (DNA methylation, histone marks, and small RNAs) encode past stress and support both seasonal priming and longer-term memory.
- Seasonal priming is closely tied to the dormancy cycle, providing a reversible, annually reset layer aligning growth and immunity with environmental cues.
- Key future challenges are to pinpoint when transient priming becomes stable stress memory and which regulatory layers truly store these ‘tree memories’.

## Open Access

Open access for this article is provided via our Subscribe to Open programme, made possible through the support of our subscribing institutions. Learn more here: https://portlandpress.com/pages/open_access_options_and_prices#conditional.

## Abbreviations

BABA: β-aminobutyric acid
C: cytosine
JA: jasmonic acid
SA: salicylic acid
